# Supplementary material for: Altered microRNA Transcriptome in Cultured Human Liver Cells upon Infection with Ebola Virus
Source: Int J Mol Sci. 2021 Apr 6;22(7):3792. doi: 10.3390/ijms22073792 (PMC8038836; doi:10.3390/ijms22073792)
Supplement: Supplementary file 1 [file ijms-22-03792-s001.zip › Supplementary_File/D_ KEGG_Analysis_Results/16-30nt_pathway_Mayinga-24h-Huh7_vs_Control-24h-Huh7_up.mature_mirna_targets/hsa_EnrichmentScore.pdf]

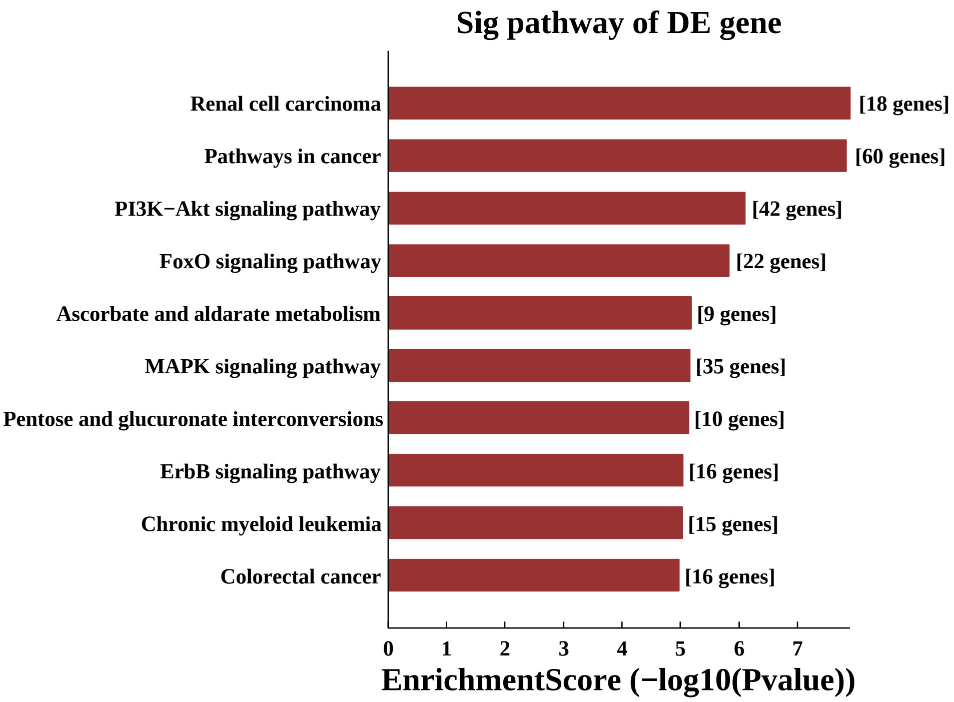

Sig pathway of DE gene
Renal cell carcinoma
Pathways in cancer
PI3K−Akt signaling pathway
FoxO signaling pathway
Ascorbate and aldarate metabolism
MAPK signaling pathway
Pentose and glucuronate interconversions
ErbB signaling pathway
Chronic myeloid leukemia
Colorectal cancer
[18 genes]
[60 genes]
[42 genes]
[22 genes]
[9 genes]
[35 genes]
[10 genes]
[16 genes]
[15 genes]
[16 genes]
0
1
2
3
4
5
6
7
EnrichmentScore (−log10(Pvalue))
